# Binge Drinking Trajectory and Decision-Making during Late Adolescence: Gender and Developmental Differences

**DOI:** 10.3389/fpsyg.2017.00783

**Published:** 2017-05-15

**Authors:** Carina Carbia, Fernando Cadaveira, Francisco Caamaño-Isorna, Socorro Rodríguez Holguín, Montserrat Corral

**Affiliations:** ^1^Department of Clinical Psychology and Psychobiology, Universidade de Santiago de CompostelaSantiago de Compostela, Spain; ^2^Consortium for Biomedical Research in Epidemiology and Public Health, Department of Preventive Medicine, University of Santiago de CompostelaSantiago de Compostela, Spain

**Keywords:** binge drinking, adolescents, alcohol, longitudinal, decision-making, IGT, gender, development

## Abstract

**Objective:** Impaired affective decision-making has been consistently related to alcohol dependence. However, less is known about decision-making and binge drinking (BD) in adolescents. The main goal of this longitudinal study was to determine the association between BD and decision-making from late adolescence to early adulthood. A second aim is to assess developmental changes and performance differences in males and females.

**Method:** An initial sample of 155 1st-year university students, (76 non-BDs, 40 females; and 79 BDs, 39 females), was followed prospectively over a 4-year period. The students were classified as stable non-BDs, stable BDs and ex-BDs according to their scores in item 3 of the AUDIT and the speed of alcohol consumption. Decision-making was assessed by the Iowa Gambling Task (IGT) three times during the study. Dependent variables were net gain and net loss. Results were analyzed using generalized linear mixed models.

**Results:** A stable BD pattern was not associated with either disadvantageous decision-making or sensitivity to loss frequency. Performance improved significantly in both genders over the study period, especially in the last blocks of the task. Females showed a higher sensitivity to loss frequency than males. No gender-related differences were observed in gains.

**Conclusion:** Performance in affective decision-making continues to improve in late adolescence, suggesting neuromaturational development in both genders. Females are more sensitive to loss frequency. Stable BD during late adolescence and emerging adulthood is not associated with deficits in decision-making. Poor performance of the IGT may be related to more severe forms of excessive alcohol consumption.

## Introduction

Adolescence is a unique period of neurodevelopment (Spear, 2013) in which the human brain undergoes significant structural and functional changes associated with progressive improvements in cognitive and affective functions (Geier and Luna, 2009; Luna, 2009; Diamond, 2013). Compared to adults, adolescents demonstrate greater reward sensitivity and heightened risk-taking behavior (Geier, 2013; Crone et al., 2016; van Duijvenvoorde et al., 2016), such as experimenting with drugs. These characteristics may be explained by asynchronous maturation of (or imbalance between) the earlier development of motivational systems and the relatively immature cognitive control (Geier, 2013; Kilford et al., 2016). In addition, as a result of ongoing neuromaturational processes, adolescence is a period of increased vulnerability to the neurotoxic effects of alcohol (Crews et al., 2007). Alcohol use by young adolescents is highly correlated with other suboptimal choices, i.e., unsafe sex (Moure-Rodriguez et al., 2016) and substance use (Windle, 2016). Binge drinking (BD) is a prevalent pattern of alcohol consumption during adolescence (Marshall, 2014). It is defined as the consumption of four drinks for women and five drinks for men in about 2 h, leading to a blood alcohol concentration (BAC) of 0.08 g/dL (National Institute of Alcohol Abuse and Alcoholism [NIAAA], 2004). A growing body of literature has documented anatomical (Squeglia et al., 2012b; Doallo et al., 2014) and functional frontal anomalies linked to BD (Squeglia et al., 2011, 2012a; Campanella et al., 2013). Cognitive deficits in young BDs has been reported, especially regarding executive functions [for a review, see (Montgomery et al., 2012; López-Caneda et al., 2014)] such as inhibitory control (Sanhueza et al., 2011) or working memory (Townshend and Duka, 2005; Scaife and Duka, 2009; Mota et al., 2013). Less attention has been paid to “hot” aspects of executive functions such as affective decision-making [linked to orbital/ventromedial prefrontal cortex (OFC/VMPC), see (Bechara, 2004; Kerr and Zelazo, 2004)]. Alcohol dependent individuals display impairments in decision-making (Verdejo-García et al., 2006; Noël et al., 2007; Brevers et al., 2014), with the severity of alcoholism associated with more pronounced deficits (Noël et al., 2007); however, little consistency has been observed in young BDs (Johnson et al., 2008; Goudriaan et al., 2011; Bø et al., 2016).

Decision-making is a complex process involving choosing between competing actions and assessing the value of short term and long term outcomes (Van den Bos et al., 2013). The Iowa Gambling Task [IGT; (Bechara et al., 1994)] was developed to measure affective decision-making under ambiguity, in which the probabilities of reward and loss are not known. Participants are told that they must gain as much money as possible by choosing cards from four virtual decks. Decks C and D are advantageous and lead to overall gain (they yield lower immediate gains but smaller losses in the long term), whereas decks A and B are disadvantageous (high immediate gains but greater losses in the long term). Decks A and B are equivalent in terms of overall losses, and decks C and D are equivalent in terms of overall gains. The decks also differ in the frequency of punishment or losses: decks A (disadvantageous) and C (advantageous) are associated with more frequent losses, although of smaller magnitude, and decks B (disadvantageous) and D (advantageous) are associated with less frequent losses of greater magnitude. Most studies have used the net gain dimension calculated simply as the total number of cards chosen from advantageous decks, or in the best case, as the preference for advantageous versus disadvantageous decks ([C+D]-[A+B]). However, fewer studies have taken into account the loss dimension represented by the relative preference for decks yielding low punishment frequency versus decks yielding high punishment frequency ([B+D]-[A+C]). This dimension has proved to be important in guiding affective decision-making (Van den Bos et al., 2013; Beitz et al., 2014; Cassotti et al., 2014). Participants must discover the rules for gains and losses by following their hunches and emotion-based signals (Damasio, 1994; Bechara, 2004; Dunn et al., 2006). The process of affective decision-making under ambiguity has been related to the ventromedial (VMPC) and orbitofrontal (OFC) prefrontal cortex, which are closely connected to the limbic system (Clark et al., 2004; Brevers et al., 2013). Healthy participants learn to prefer long term advantageous decks associated with immediate moderate rewards over long-term disadvantageous decks with immediate high rewards. By contrast, patients with ventromedial prefrontal (VM) cortex lesions often make decisions based only on the immediate consequences (Bechara et al., 1994).

Previous studies using the IGT, have shown disadvantageous performance of decision-making tasks by Chinese adolescent BDs relative to occasional (Xiao et al., 2009) and never drinkers (Johnson et al., 2008; Xiao et al., 2012). Similar findings have recently been reported for Korean BDs (Yoo and Kim, 2016), who also selected more cards than non-BDs from the disadvantageous deck B. Goudriaan et al. (2007) reported that poor decision-making was observed in adolescent “chronic high-BDs” compared with “low BDs.” Another study by the same group (Goudriaan et al., 2011), observed that poor performance of the IGT was predictive of BD in male but not in female adolescents, which may be explained by the fact that males undertook more BD episodes and consumed more quantity of alcohol than females. In young adults with less extreme patterns of alcohol consumption, BD was associated with differences in performance in the loss dimension but not in the gain dimension (Bø et al., 2016). As far as we are aware, no studies to date have addressed this relationship with a longitudinal design involving repeated measures of decision-making performance during adolescence. The influence of potential confounding factors, such as substance use, psychopathological symptoms, variations in the definition of BD and possible cultural influences, has also been poorly considered. The fact that some studies only took into account the gain dimension and did not control for general executive measures (i.e., working memory or inhibition) are possible limitations, leading to an incomplete comprehension of affective decision-making in adolescent BDs.

The ability to select progressively from the advantageous decks continues to develop during adolescence (Hooper et al., 2004; Cassotti et al., 2011), and even during young adulthood (Cauffman et al., 2010). Children and adolescents also seem to choose cards with infrequent losses. This tendency, also referred to as frequency bias, decreases with age (Huizenga et al., 2007; Cassotti et al., 2011, 2014). Gender differences in developmental trajectories and performance of the IGT are poorly understood. There is no broad agreement about how males and females differ in gain and loss dimensions. Some studies have reported that males outperform females in gains (Overman and Pierce, 2013; Evans and Hampson, 2015), while others propose that both are equally capable of choosing from advantageous decks but that females are more sensitive to loss frequency (Hooper et al., 2004; Van den Bos et al., 2013).

Thus, the main aim of this study was to determine whether a trajectory of stable BD in healthy university students is associated with disadvantageous decision-making. A further aim was to assess the developmental trajectories during emerging adulthood (18–23 years old) in decision-making, in each gender separately, and taking into account gain and loss dimensions. We considered the following hypotheses: (I) stable BDs will display disadvantageous decision-making relative to age-matched stable non-BDs, (II) males and females will perform equally in net gains, but females will present a stronger frequency bias than males; and (III) both females and males will show improvements in performance during late adolescence.

## Materials and Methods

### Participants

Participants were selected through an anonymous questionnaire administered in class [see (Caamaño-Isorna et al., 2008) for more details]. The questionnaire included the Alcohol Use Disorders Identification Test (AUDIT) (Babor et al., 2001) and questions related to alcohol use such as speed of consumption and age of drinking onset. A BD episode was defined as consumption of six drinks at a speed of more than two drinks per hour, bringing the BAC to 0.8 g/l or higher. A standard drink unit of ethanol varies across countries: thus, while in Spain it is defined as 10 g of ethanol, in e.g., USA, it is 14 g. The classification criteria were based on the students’ responses to two questions: the third item of the AUDIT (How often do you have six or more drinks on a single occasion? Never/Less than Monthly/Monthly/Weekly/Daily or almost daily) and one question related to the speed of consumption measured as drinks per hour. BDs consumed six drinks on one occasion monthly or weekly, and the speed of alcohol consumption was three drinks or more per hour. The non-BDs were defined as those who never consumed six drinks on one occasion (or less than monthly) and who consumed alcohol at a speed of two drinks or less per hour.

As the objective of this study was to assess the BD trajectory, the sample was classified as stable non-BDs (those who remained as controls during the assessment period), stable BDs (who remained as BDs during the assessment period) and ex-BDs (those who abandoned the BD pattern at the first or second follow-up and remained with non-BD consumption thereafter). Abstainers were not included in the study. The classification criteria did not allow transitions in the trajectories (e.g., a non-BD who changed to a BD at the second evaluation would be excluded from the analysis in the last evaluation but maintained for the previous evaluations). The number of participants decreased throughout the study: 155 participants at baseline (76 non-BDs, 40 females; and 79 BDs, 39 females); 93 at the first follow-up (39 stable non-BDs, 21 females; 33 stable BDs, 14 females, and 21 ex-BDs, 15 females); and 74 at the final follow-up (33 stable non-BD participants, 18 females; 17 stable BDs, 8 females and 24 ex-BDs, 15 females). Each alcohol consumption trajectory included the following number of total data points: 148 stable non-BDs, 129 stable BDs, and 45 ex-BDs. The trajectory of performance in each gender was computed with a total number of 170 data points for females (79 at baseline, 50 at first follow-up, and 41 at second follow-up) and 152 for males (76 at baseline, 43 at first follow-up, and 33 at second follow-up).

### Procedure

After being classified according to alcohol consumption, participants were interviewed to obtain clinical and sociodemographic information. To reduce potentially confounding factors, several exclusion criteria were used: personal history of neurological disorders; history of psychopathology (DSM-IV-TR) such as attention-deficit hyperactivity disorder or conduct disorder; current psychopathological symptoms as assessed by the Symptom Checklist-90-R (SCL-90-R) (Degoratis, 1983) (participants were excluded if they had scores above 90th in the Global Severity Index [GSI] or in at least two symptomatic dimensions); consumption of other drugs, except nicotine and cannabis (sporadic cannabis users and smokers were not excluded). None of the participants included in the study consumed cannabis daily. Other exclusion criteria included diagnosis of alcohol use disorders, severe non-corrected motor or sensory deficits, family history of alcoholism in first- and second-degree relatives, and other major psychopathological disorder (depression, anxiety, schizophrenia diagnosis etc.) in first-degree relatives. All three evaluations were made on average every 22 months. In each, a neuropsychological battery was administered together with an interview in which the same exclusionary criteria were considered in order to yield a sample of university students with no other risk factors. Only those participants who attended the previous evaluation (and met the inclusion criteria) were contacted again for each new evaluation. This implies that participants who underwent the final evaluation had also undergone all previous assessments. All participants received some monetary compensation and gave written informed consent in accordance with the Declaration of Helsinki. This research was approved by the bioethics committee of University of Santiago de Compostela.

### Material

*Iowa Gambling Task* (Bechara et al., 1994): The IGT is a computerized version of the gambling task. In this task, individuals are invited to choose a card from four virtual decks of cards presented on a screen and labeled A, B, C, and D. The aim of the task is to earn as much money as possible. The characteristics of the decks are not disclosed and must be inferred gradually on the basis of positive and negative feedback. When the subject selects a card, a message indicating the amount of money won or lost is displayed on the screen. Decks C and D are advantageous and lead to overall gain (lower immediate gains but smaller losses in the long run), whereas A and B are disadvantageous (high immediate gains but greater losses in the long run). Decks A and B are equivalent in terms of overall net losses, and decks C and D are equivalent in terms of overall net gains. The decks also differ in the frequency of loss or punishment, with decks A (disadvantageous) and C (advantageous) having more frequent punishments but of smaller magnitude and decks B (disadvantageous) and D (advantageous) having less frequent punishments but greater magnitude. The task consists of 5 blocks of 20 cards, i.e., a total of 100 cards. The net gain dimension represents the relative preference for advantageous versus disadvantageous decks ([C+D]-[A+B]). The net loss dimension is the relative preference for low punishment frequency decks versus high punishment frequency decks ([B+D]-[A+C]).

*Self-Ordered Pointing Test, abstract design version (SOPT)* (Petrides and Milner, 1982): This test consists of a booklet of abstract designs repeated on all pages but with a different position on each new page. The participant is asked to point out a different stimulus on each page without repeating previous choices. The test is divided into four blocks of increasing difficulty (6, 8, 10, and 12 stimuli), and each block consists of three trials. The total number of errors was recorded for each participant. The SOPT assesses planning and self-monitoring aspects of working memory. The scores in the SOPT allow us to control the possible interference of working memory deficits in decision-making.

### Statistical Analysis

Generalized linear mixed models (GLMMs), in which maximum log-likelihood was approximated by adaptive Gauss-Hermite quadrature, were used in the statistical analysis (Brown and Prescott, 2014). GLMMs allow analysis of repeated measurements (measurement correlation and intra-individual heterogeneity) with greater statistical power than classical regression models (Gibbons et al., 2010). Unlike other repeated measures analysis, GLMMs can handle a different number of participants in each evaluation. All analyses were performed using the free R (version 3.1.1) statistical software environment (R Core Team, 2015) with the lme4 package (Bates et al., 2014), and all results were expressed as relative risks (RRs) and their 95% confidence intervals (CIs). This type of coefficient requires reference categories in order to establish the comparisons. Values higher than one with significant intervals are indicative of a good performance for gain, whereas values below one reflect less frequency bias for loss.

To construct the models, we used net gain and net loss (over 100 trials and in each block) as dependent variables, with individual observations as level 1 and students as level 2; random effects among students were considered to control initial intra-individual heterogeneity. In order to avoid negative scores, a constant value of 100 was summed to gains and losses. Different models were constructed for females and males in order to assess any developmental changes. The effect of alcohol consumption trajectory and possible interactions with time and gender were modeled. Frequency of cannabis use, age of drinking onset and the GSI score of the SCL-90-R were tested to determine whether they had explanatory roles. The independent variables with a statistical significance lower than 0.2 at a bivariate level were included in the multivariate models. The non-significant independent variables were eliminated from this maximum model when the coefficients of the main exposure variables did not vary by more than 10% and the value of Schwartz’s Bayesian Information Criterion (BIC) decreased. The number of errors in the SOPT was used to control the effect of possible working memory deficits. Finally, we used JASP statistical software (JASP Team, 2016) to perform complementary Bayesian independent sample *t*-tests (by time and group), for null-hypothesis significance testing (Masson, 2011).

In order to ensure that the classification of stable trajectories of consumption (e.g., a non-BD who changed to a BD at the second evaluation would be excluded from the analysis in the last evaluation but maintained for the previous evaluations) did not have any relevant influence on the results, we performed the same statistical analysis allowing transitions in consumption trajectory. For example, a non-BD in the first evaluation who changed to a BD in the second assessment was then considered within this new group at that specific time point. In other words, the statistical model considered the specific pattern of consumption at each time point, thus reducing the sample attrition over time. However, the results obtained were almost identical. We therefore used the stable trajectory classification, for the sake of simplicity.

## Results

### Demographic, Substance Use Variables and Performance

The descriptive characteristics of the sample at baseline are shown in **Table 1**. Groups differed in the following variables: age of onset of alcohol use, *t*(137) = 4.83, *p* = 0.001; total AUDIT scores, *t*(124.32) = 15.68, *p* = 0.001; number of drinks per hour, *t*(153) = 14.48, *p* = 0.001; grams of alcohol consumed during the week, *t*(73.61) = 8.44, *p* = 0.001, and grams of alcohol consumed on the day of highest consumption, *t*(71.51) = 5.94, *p* = 0.001. There were no differences in psychopathological symptoms measured by GSI scores of SCL-90-R test, *t*(153) = 0.76, *p* = 0.447. Groups differed in age, *t*(152) = 2.86, *p* = 0.005, the BDs were slightly older than the non-BDs. Group differences were also found in cannabis use, *X*^2^(2, *N* = 153) = 19.50, *p* = 0.001, and tobacco use, *X*^2^(2, *N* = 153) = 8.12, *p* = 0.004. The groups did not differ in estimated intellectual level as assessed by the Vocabulary subtest (WAIS-III) (Wechsler, 1997). Means and standard deviations for net gain and net loss over time in each trajectory and gender are shown in **Table 2**. **Table 3** depicts how the different trajectories of alcohol consumption performed throughout the task (means by block), with progressively more advantageous cards being chosen.

**Table 1:** Group means (standard deviation) for demographic and clinical data at baseline.

	Non-BDs (*n* = 76)	BDs (*n* = 79)
Sex (m/f)	36/40	40/39
Age^∗∗^	18.58 (0.60)	18.87 (0.63)
Age of onset alcohol use^∗∗∗^	15.78 (1.04)	14.8 (1.30)
AUDIT total^∗∗∗^	2.95 (2.58)	12.22 (4.55)
Number of drinks per hour^∗∗∗^	1.04 (0.84)	3.39 (1.14)
Alcohol (g) consumed during the week^a∗∗∗^	42.19 (52.79)	302.46 (251.13)
Alcohol (g) consumed on the day of highest consumption^a∗∗∗^	27.63 (31.93)	166.69 (192.12)
Occasional cannabis users^∗∗∗^	0	8
Occasional smokers^∗∗^	3	24
GSI (SCL-90-R), Pc	46.39 (28.83)	50.09 (31.36)
WAIS-III Vocabulary	12.56 (1.97)	12.11 (1.62)

*^a^The week prior to the evaluation. ^∗∗^*p* < 0.01, ^∗∗∗^*p* < 0.001.*

**Table 2:** Means (and standard deviations) for net gain and loss over time.

	Baseline		First follow-up		Second follow-up	
	Gain	Loss	Gain	Loss	Gain	Loss
Females	15.60 (22.83)	30.05 (23.90)	21.69 (25.19)	28.46 (22.51)	33.44 (22.22)	21.05 (25.66)
Males	5.00 (22.31)	10.8 (23.81)	30.12 (28.35)	11.98 (28.46)	37.54 (31.15)	14.44 (31.72)
Stable non-BDs	10.64 (22.03)	18.45 (27.58)	31.95 (23.17)	13.76 (31.17)	36.37 (22.37)	21.97 (33.02)
Stable BDs	11.15 (25.22)	25.70 (22.84)	27.06 (26.05)	18.81 (23.83)	41.86 (21.29)	7.00 (26.51)
Ex-BDs			21.33 (28.33)	29.43 (21.29)	25.83 (27.67)	17.35 (25.24)

*Gain = ([C+D]-[A+B]); Loss = ([B+D]-[A+C]).*

**Table 3:** Means (and standard deviations) for gain in each block of the IGT.

	Stable non-BDs	Stable BDs	Ex-BDs
**Overall performance^a^**			
Gain block 1	-1.86 (6.52)	-1.87 (4.12)	-1.36 (7.05)
Gain block 2	2.79 (6.89)	2.99 (7.52)	3.09 (6.70)
Gain block 3	6.45 (8.24)	5.89 (8.19)	6.00 (8.51)
Gain block 4	7.89 (8.12)	8.33 (8.75)	7.45 (7.70)
Gain block 5	9.69 (8.48)	7.71 (9.24)	8.5 (8.64)
**Second follow-up^b^**			
Gain block 1	-3.00 (6.52)	0.00 (5.14)	-1.48 (8.18)
Gain block 2	6.00 (8.35)	4.29 (8.26)	3.04 (7.18)
Gain block 3	9.94 (8.07)	10.71 (6.16)	6.18 (9.46)
Gain block 4	12.06 (7.39)	12.86 (6.41)	7.48 (7.70)
Gain block 5	11.38 (8.58)	14.00 (7.23)	10.61 (7.30)

*Gain = ([C+D]-[A+B]); Loss = ([B+D]-A+C]). ^a^Average performance in the three assessments. ^b^Average performance after 4 years of follow-up.*

### Gender-Related Differences in Decision-Making

Females and males did not differ in relation to net gain (RR = 0.98, 95% CI [0.90, 1.06], *p* = 0.595) nor in any particular block in this dimension. However, for net loss females showed a 12% RR (1.12, 95% CI [1.03, 1.20], *p* = 0.005) of selecting more cards with a low frequency loss (frequency bias) relative to males. When considering the effect on blocks, males and females performed similarly in loss in the first three blocks of the task. The frequency bias was notable in the last two blocks, i.e., blocks four (RR = 1.03, 95% CI [1.01, 1.06], *p* = 0.046]) and five (RR = 1.05, 96% CI [1.01, 1.09], *p* = 0.006). This means that females showed a 5% risk of being guided by frequency of loss in the last block in comparison with males. Thus, females chose more decks with low frequency of punishment to a greater extent than males in total, and this effect was particularly evident in the last part of the task.

With respect to deck preferences, deck C was the most frequently chosen by both genders, followed by deck D (both of these are advantageous decks) and then B; deck A was chosen least often. Females chose significantly fewer cards from deck C in 100 trials (RR = 0.83, 95% CI [0.71, 0.98], *p* = 0.032) in comparison with males, more specifically 20% (1/0.83 = RR 1.20) fewer than chosen by males.

### Developmental Changes in Decision-Making by Gender

Both females and males showed improvements on the IGT in net gain. However, only females improved in net loss (**Table 4**). Regarding net gain, females showed a significant improvement at the first follow-up (RR = 1.12, 95% CI [1.07, 1.18], *p* < 0.001) and second follow-up (RR = 1.20, 95% CI [1.13, 1.27], *p* < 0.001) relative to baseline. This indicates that at the second follow-up performance of the task was 20% better as females chose more advantageous cards than in baseline. It should be noted that values higher than one with significant intervals are indicative of a good performance in gain. The improvement at the second follow-up was also significantly different from the performance at the first follow-up (RR = 1.07, 95[1.02, 1.11], *p* = 0.002, but smaller (7%). Males also showed a significant improvement at the first follow-up (RR = 1.30, 95% CI [1.22, 1.38], *p* < 0.001) and the second follow-up (RR = 1.33, 95% CI [1.25, 1.43], *p* < 0.001) relative to baseline. However, there were no significant changes between first and second follow-up (RR = 1.00, 95% CI [0.95, 1.05], *p* = 0.906). Thus, the improvement shown by males (30%) was limited to the first follow-up, while females continued to improve until the second follow-up.

**Table 4:** Developmental changes in females and males for gain and loss in each block.

	GLMMs. Relative Risk. 95% CI			
	Females		Males	
	First follow-up^a^	Second follow-up^a^	First follow-up^a^	Second follow-up^a^
**Net Gain**	1.12 [1.07, 1.18]^∗∗∗^	1.20 [1.13, 1.27]^∗∗∗^	1.30 [1.25, 1.43]^∗∗∗^	1.33 [1.25, 1.43]^∗∗∗^
1° Block	0.99 [0.94, 1.04]	0.99 [0.95, 1.06]	1.02 [0.97, 1.09]	1.02 [0.95, 1.08]
2° Block	1.02 [0.97, 1.07]	1.04 [0.99, 1.10]	1.05 [0.99, 1.12]	1.04 [0.98, 1.11]
3° Block	1.05 [0.99, 1.10]	1.07 [1.02, 1.13]^∗∗^	1.08 [1.02, 1.15]^∗∗^	1.09 [1.02, 1.16]^∗∗^
4° Block	1.03 [0.98, 1.08]	1.06 [1.00, 1.11]^∗^	1.09 [1.03, 1.15]^∗∗^	1.10 [1.03, 1.17]^∗∗^
5° Block	1.04 [0.99, 1.09]	1.07 [1.01, 1.12]^∗^	1.06 [1.00, 1.12]^∗^	1.07 [1.00, 1.14]^∗^
**Net Loss**	0.95 [0.90, 0.99]^∗^	0.88 [0.84, 0.93]^∗∗∗^	0.98 [0.92, 1.04]	0.98 [0.91, 1.05]
1° Block	1.00 [0.95, 1.05]	1.00 [0.95, 1.05]	1.00 [0.94, 1.06]	0.98 [0.92, 1.05]
2° Block	1.00 [0.95, 1.05]	0.98 [0.93, 1.03]	1.03 [0.98, 1.10]	1.04 [0.98, 1.11]
3° Block	1.01 [0.96, 1.06]	0.98 [0.93, 1.04]	0.99 [0.94, 1.05]	0.99 [0.93, 1.05]
4° Block	0.98 [0.93, 1.03]	0.95 [0.90, 1.01]	0.99 [0.93, 1.05]	0.99 [0.93, 1.06]
5° Block	0.96 [0.91, 1.00]	0.95 [0.90, 0.99]^∗^	0.92 [0.87, 0.98]^∗^	0.94 [0.88, 1.01]

*GLMMs, generalized linear mixed models. CI, confidence intervals. ^a^Reference category = baseline. Values higher than one with significant intervals are indicative of a good performance in gain whereas in loss values below one reflect less frequency bias. ^∗^*p* < 0.05, ^∗∗^*p* < 0.01, ^∗∗∗^*p* < 0.001.*

In relation to net loss (also in **Table 4**), females showed an improvement at the first follow-up (RR = 0.95, 95% CI, [0.90, 0.99], *p* = 0.049) and the second follow-up (RR = 0.88, 95% CI [0.84, 0.93], *p* < 0.001), and the changes in performance between the first and second follow-up were also significant (RR = 0.92, 95% CI [0.88, 0.96], *p* < 0.001). Values below one reflect less frequency bias. In other words, females showed an improvement of 5% (1/0.95, RR 1.05) in net loss at the first follow-up and improvement of 14% (1/88 = RR 1.14) at the second follow-up relative to baseline. Conversely, males did not show any significant changes in net loss over time.

When considering individual blocks, females presented significant improvements in blocks 3, 4, and 5 in net gain at the second follow-up relative to baseline {e.g., an improvement of 7% in block 5 (RR = 1.07, 95% CI [1.01, 1.12], *p* = 0.017)}. Males showed an improvement in the same blocks for net gain at the first follow-up. Although the latter improvement was maintained in the second follow-up (as shown in **Table 4**), there were no additional improvements. This implies that no significant changes between the first and second follow-up were observed in males on gain blocks. In net loss, females showed an improvement of 5% in block 5 (RR = 0.95, 95% CI [0.90, 0.99], *p* = 0.042) at the second follow-up relative to baseline. Males also showed an improvement in the same final block of the task (RR = 0.92, 95% CI [0.87, 0.98], *p* = 0.012), although earlier than females (i.e., at first follow-up).

### Binge Drinking during Adolescence

In the IGT, stable BDs performed similarly to stable non-BDs in relation to net gain (RR = 0.95, 95% CI [0.83, 1.08], *p* = 0.447) and net loss (RR = 1.07, 95% CI [0.93, 1.23], *p* = 0.322) and controlling for working memory (number of errors in the SOPT) and age of drinking onset (**Table 5**). Although entering the final model (*p <* 0.2 at the bivariate level), the number of errors in the SOPT and age of onset were not significantly associated with IGT performance. No effects were observed when considering the different blocks of the task individually. Ex-BDs also did not differ significantly from non-BDs participants in the task. No interactions between the pattern of consumption and gender were observed. Frequency of cannabis use and psychopathological symptoms (GSI score of the SCL-90-R) were not significantly associated with performance of the IGT in the bivariate/multivariate models. Complementary Bayesian analysis for null-hypothesis significance showed evidence supporting the null hypothesis (e.g., Bayes factor [BF10] of 0.176 at baseline for the comparison of net gain between stable non-BDs and stable BDs and BF10 of 0.194 at the last follow-up).

**Table 5:** Relationship between binge drinking trajectory and decision-making.

	GLMM. Relative Risk, 95% CIs	
	Stable BDs^a^	Ex-BDs^a^
Net Gain	0.95 [0.83, 1.08]	1.01 [0.86, 1.18]
Net Loss	1.07 [0.93, 1.23]	1.02 [0.87, 1.20]

*GLMMs, generalized linear mixed models. CIs, confidence intervals. ^a^Reference category = stable non-BDs.*

## Discussion

The main aim of this study was to determine whether a stable BD trajectory was associated with disadvantageous decision-making in healthy university students. Contrary to our hypothesis, a stable pattern of BD throughout late adolescence (18–23 years old) was not associated with poor performance of the IGT. A further aim was to analyze the developmental changes in decision-making during this period and examine differences between females and males in performance of the IGT. Females and males performed equally well in net gain, indicating that both genders were capable of choosing advantageous decks that yield good long term results. However, as we hypothesized, females were more sensitive to loss frequency, i.e., they chose more cards from decks with low loss frequency than males did. This frequency bias was particularly evident in the final blocks of the task and in long-term advantageous decks, as indicated by females choosing significantly fewer cards from deck C (advantageous deck with high frequency loss) than males. Thus, females seem to focus both on long-term advantageous decks and frequency of punishment, which is a rather unsuccessful strategy in this task.

In line with our findings, a developmental study with adolescents observed a stronger frequency bias in females than in males despite both having equivalent performance in gains (Hooper et al., 2004). Similarly, another study found that over 100 trials males and females performed similarly in gains, and both were able to solve the task efficiently choosing more advantageous cards over disadvantageous ones (Van den Bos et al., 2013). Females were more sensitive to losses than males, especially in the long-term advantageous decks, as observed in the present study. According to the authors, females attend to two different aspects of the task – frequency of loss and the long-term pay off – while men only attend to the latter (Van den Bos et al., 2013). Conversely, some studies have found that males outperform females in net gains (Evans and Hampson, 2015). Although the meaning of gender-related differences on IGT performance it is far from clear, the involvement of some neurobiological differences has been suggested (Overman and Pierce, 2013; Van den Bos et al., 2013). In a study using positron emission tomography (PET), men performed better on the task (measured as cards from advantageous decks minus cards from disadvantageous decks) and showed greater lateralized brain activity in the right hemisphere than women (Bolla et al., 2004). This finding may be associated with gender-related differences in processing information, i.e., men tend to be more right-oriented (global information) and woman more left-oriented (detailed information), as explained in Van den Bos et al. (2013). The present results might be consistent with the above as females seem to focus on detailed aspects of the task (long term advantageous decks and frequency of loss) rather than the global outcome (gains in long term advantageous decks).

Secondly, as we expected, both genders showed improvements in performance during emerging adulthood in gain. The improvement in net gain was evident in the final blocks of the tasks but not at the beginning, which might suggest neuromaturational developmental rather than simple practice effects. The final blocks of the task seem to involve different cognitive requirements than the first part, probably involving “cold” executive process to a greater extent (Noël et al., 2007; Brevers et al., 2014). Females showed improvements in net gain over a longer time (until a later age) than males, although this probably reflects more opportunity for improvement due to the relatively poor initial performance (stronger frequency bias at baseline) in this task. Regarding net loss, the frequency bias decreased in females over time. However, males did not show any changes in loss over time, probably because this dimension is not as relevant in their performance as in females. These findings parallel previous studies showing that the ability to select progressively from the “good” decks on the IGT continues to improve not only during adolescence (Hooper et al., 2004; Cassotti et al., 2011) but also during early adulthood (Cauffman et al., 2010) and that the frequency bias decreased with age (Huizenga et al., 2007; Cassotti et al., 2011, 2014).

Finally, stable BD throughout the university years was not associated with poor performance of the IGT. Stable BDs and ex-BDs performed similarly to stable non-BDs regarding gain and loss, considering both net scores and individual blocks. Likewise, Bø et al. (2016) found that the BD score of young adults was not predictive of difficulties in choosing from advantageous decks on the IGT. However, heavy drinking was associated with selecting more cards from decks with frequent losses (only in the first 40 trials). The authors of the study calculated the frequency of loss as decks ([A+D]-[B-C]), which to our view, does not clearly account for high versus low frequency of punishment. In a recent study (Yoo and Kim, 2016), Korean student BDs selected more cards from deck B and showed disadvantageous decision making (they chose more cards from decks A and B) relative to non-BDs, particularly in the third and fourth block. The loss dimension was not analyzed, and working memory- or a general executive function score- was not accounted for. In addition, BD participants had to score between 12 and 26 in the AUDIT for inclusion in the study. Thus, the level of alcohol consumption may have been higher in this sample than in our sample, i.e., a cut-off of >20 warrants diagnostic evaluation for alcohol dependence, as indicated in the AUDIT guidelines (Babor et al., 2001). Johnson et al. (2008) found that Chinese adolescent BDs showed disadvantageous decision-making relative to “never-drinkers” in the last 50 trials. Interestingly, comparison of BDs with adolescent “ever drinkers” (a group with similar characteristics to the non-BDs in the present study) did not reveal any differences in performance, similarly to our findings. The same was observed in the comparison between BDs and “past 30 days drinkers” (a group with more drinking problems than “ever drinkers”). Two studies by the same research group showed that performance of the IGT by Chinese adolescent BDs (only three females were consistent BDs) was poorer than in occasional drinkers (Xiao et al., 2009) and found higher activity in the left amygdala and insula bilaterally -regions that form part of the neural circuitry involved in affective decision-making- in BDs relative to never drinkers (Xiao et al., 2012). No differences in performance between males and females were reported in these three previous studies with Chinese adolescents or in the Korean sample (Yoo and Kim, 2016). In this respect, the extent to which cultural differences in the IGT may influence task performance requires further study (Singh and Khan, 2012).

Another study based in the US reported disadvantageous IGT performance in chronic high-BDs relative to low-BDs, although working memory was not controlled for Goudriaan et al. (2007). Age of drinking onset or the age of the first time being drunk was not predictive of IGT performance. The authors reported that females showed a frequency bias. In this study some of the participants, particularly high-BDs, were diagnosed with both alcohol and cannabis abuse/dependency as well as other DSM-IV diagnoses [e.g., antisocial personality disorder which has been associated with poor IGT performance (Miranda et al., 2009)]. Goudriaan et al. (2011) showed that disadvantageous decision-making may be a predictor of heavy alcohol use. Poor performance of the IGT (percentage of cards form advantageous decks) was predictive of high levels of heavy drinking in male but not in female adolescents. The fact that men reported heavier alcohol use than women may explain this gender interaction – women had lower scores both on the quantity/frequency of alcohol use and fewer BD episodes. Inhibitory control -measured by a stop signal task- was not predictive of heavy drinking, when baseline alcohol use was controlled for. The last two studies only analyzed the first 80 trials of the task because of an artifact in the data, which is a possible constraint.

Together, the above-mention studies have shown little consistency, possible due to the previous considerations (e.g., psychiatric disorders, methodological issues). Overall, it seems that poor decision-making is associated with high levels of heavy drinking, as occurs in more severe forms of alcohol consumption such as alcohol dependence (Brevers et al., 2014). To our knowledge, this is the first longitudinal study assessing the relationship between BD and decision-making- involving repeated measures of the IGT – in young adults with no other risk factors. Our findings indicate that a less severe pattern of BD is not related to impairments in decision-making in university students. Further studies using other executive tasks and considering BD trajectories with different levels of consumption and taking into account both gain and loss dimensions are needed to confirm these results. In addition, increasing the number of IGT trials [as suggested in Brevers et al. (2014)] may be useful to determine specific decision-making deficits. The IGT is a complex task that may involve different cognitive and affective processes at the beginning of the task (exploration guided by emotion or intuition) than in the last part (some knowledge about probabilities; executive functions). For instance, Noël et al. (2007) found that alcoholic participants who had recently undergone detoxification displayed poorer performance of the last 20 trials of the IGT and other executive tasks (inhibition of prepotent responses, manipulation of information stored in working memory etc.). Response inhibition was the best predictor of impaired performance in the last part of the IGT. Thus, this modification may be helpful for identifying subtle executive difficulties, especially in a population such as university student BDs with no other risk factors. Furthermore, normal participants seemed to keep improving their performance when another set of 100 cards was added at the end of the first 100 trials (Overman and Pierce, 2013), which according the authors may indicate that the process of decision making is not fully complete at the end of the original version. In our case, this may serve to identify possible “slow learners” in relation to excessive alcohol consumption.

One possible limitation of this study is the sample attrition. This mainly affects the analysis of progression over time (each follow-up relative to baseline) and especially the last assessment. GLMMs offer the advantage of being able to handle different number of participants in each evaluation. Thus, a participant who has just two assessments is included in the analysis until that point. Therefore, the findings related to overall performance in males versus females or the trajectories of consumption are less affected by this limitation, as a greater number of data points are included. Besides, these models also consider the response correlation in repeated measures – i.e., correlated measurement errors and heterogeneity of participants - resulting in greater statistical power (Gibbons et al., 2010). Another potential limitation is the fact that practice effects may represent a confounding factor in the interpretation of developmental improvements, as the same version of the IGT was used for all the assessments. However, the assessments were made on average every 2 years and the characteristics of the decks were not disclosed. Indeed, participants did not show any improvements over time in the first part of the task (40 first trials). To our view, these findings suggest that knowledge accumulated from previous evaluations does not substantially help participants to perform the task. In other words, the first trials seem to be as difficult as at baseline, with “an exploratory phase” remaining, despite some familiarity with the general procedure.

## Conclusion

Decision-making -as assessed by IGT performance- seems to continue to improve in late adolescence. Both genders are equally capable of learning throughout the task, preferring advantageous over disadvantageous decks. However, females are more sensitive to loss frequency than males. Finally, healthy university students with a stable BD trajectory performed similarly in gain and loss dimensions on the IGT relative to age-matched non-BDs. In view of the above, disadvantageous performance in decision-making under ambiguity may be associated with more severe or extreme forms of heavy drinking.

## Author Contributions

CC, MC, FC-I, FC, and SR, participate revising it critically for important intellectual content. CC, FC-I, FC, SR, and MC, made substantial contributions to conception and design, and/or acquisition of data, and/or analysis and interpretation of data. All gave final approval of the manuscript.

## Conflict of Interest Statement

The authors declare that the research was conducted in the absence of any commercial or financial relationships that could be construed as a potential conflict of interest.
